# Metagenomics analysis of the morphological aspects and bacterial composition of broiler feces

**DOI:** 10.1016/j.psj.2022.102401

**Published:** 2022-12-09

**Authors:** Bauer O. Alvarenga, Jacqueline B. Paiva, Andrei I.S. Souza, Denise R. Rodrigues, Polyana C. Tizioto, Antonio J. Piantino Ferreira

**Affiliations:** ⁎ Biocamp Laboratories Ltda., Campinas, Brazil; † Department of Inspection of Animal Products, Ministry of Agriculture, Livestock and Food Supply (MAPA), Brasília, Brazil; ‡ NGS Genomics Solutions, São Carlos, Brazil; § School of Veterinary Medicine and Animal Science of University of São Paulo, São Paulo, Brazil

**Keywords:** fecal morphology, fecal microbiota, metagenomic, cecal discharge

## Abstract

In this descriptive study, we used metagenomics to analyze the relationship between the morphological aspects of chicken feces and its respective bacterial compositions. The microbiota composition was determined by sequencing the V4 region of the 16S rRNA genes collected from fresh broiler feces at 19 d old. In total, 48 samples were collected and divided into 8 groups of 6 samples each. The morphological changes studied were feed passage (**FP**) and reddish mucus (**RM**). Each was classified into 3 levels of intensity: 1 (slight), 2 (moderate), or 3 (intense). Thus, the 8 groups studied were feed passage (**FP-1; FP-2; FP-3**), reddish mucus (**RM-1; RM-2; RM-3**), normal ileal feces (**NIF**), and cecal discharge (**CD**). The alpha diversity (Shannon's index) revealed that the CD group showed greater diversity, and was significantly different from FP-2, FP-3, and RM-1. The beta diversity showed that the CD group samples were more homogeneous than the ileal feces groups. The relative abundance analysis revealed that *Firmicutes* and *Proteobacteria* were the most abundant phyla in the ileal feces groups. In CD, *Firmicutes* and *Bacteroidetes* were the most abundant. The relative abundance at the genus level revealed 136 different bacterial genera. In the ileal feces groups, the two most abundant genera were *Lactobacillus* and *Escherichia/Shigella*, except in the FP-1 and RM-2 groups, which had the opposite order. Unlike the others, the CD group had a higher abundance of *Bacteroides* and *Faecalibacterium*. When comparing the NIF group with the others, significant changes were found in the fecal microbiota, with nine genera for the FP groups, 19 for the RM groups, and 61 when compared to CD. The results of the present study suggest that evaluation of fecal morphology is a fundamental task that makes it possible to act quickly and assertively, as the morphological aspects of the feces may be related to the composition and structure of fecal microbiota.

## INTRODUCTION

The interaction between the commensal microbiota and the host organism results in a symbiotic relationship (Malard et al., 2021). Specifically, in birds, Apajalahti et al. (2004) revealed that the ileal microbiota has 10^9^ colony forming units (**CFU**)/g content, while in the cecum, this value is higher, reaching 10^11^ CFU/g content. Thus, abundance and diversity vary along the intestinal segments, and the microbiota perform essential functions to the host's health, as they are related to the maintenance of intestinal integrity, defense against pathogens, nutrient production, and stimulation of immunity, became imperious the balance of the gut microbiota (Gaskins, 2000; Van der Wielen et al., 2000; Snel et al., 2002; Oviedo-Rondon et al., 2006; Rinttilä and Apajalahti, 2013; De Vadder et al., 2014).

Analyses of gut microbiota have generally been performed by sampling the gut contents of birds, requiring the sacrifice of animals. However, in this study, we used a noninvasive method, as we sampled only feces, and it was not necessary to sacrifice any birds. According to Stanley et al. (2015), there is a high correlation between the fecal microbiota and the cecal microbiota of birds. Because it is a noninvasive method, it is possible to sample feces from the same bird at different ages.

Analysis of the morphological aspects of broiler feces is an important task that should be part of the poultry companies’ routine because there is a relationship between enteric bacteria and the morphological aspects of feces (Li and Ding, 2010).

Commonly, chickens can eliminate two types of feces, ileal droppings and cecal discharges. The ileal droppings do not pass through the cecum, may contain a portion of urine, and are eliminated several times a day. On the other hand, cecal discharges are eliminated through the cecum once or twice a day and without the presence of urine Changes such as feed passage, reddish mucus, the presence of foamy and watery feces, mucus, or color change (yellowish or greenish) are undesirable characteristics in broiler feces. Such changes can occur at different intensities and can be caused by several factors (Ito et al., 2009).

The intestinal functional differences are linked to the feed digestion process and absorption of nutrients; since the feed goes through the intestine, it changes because of the digestive process. Likewise, the intestinal microbiota also differs along the intestinal segments (Franzosa et al., 2015). Although the emergence of 16S rRNA sequencing and metagenomics pipelines has promoted deep insights into the intestinal microbiota taxonomic profile in broilers, there is still a lack of information about the link between the morphological aspects of broiler feces and its bacterial composition.

This descriptive study employed metagenomics analysis to evaluate the bacterial composition of broiler feces with varying degrees of feed passage or the presence of reddish mucus compared to normal ileal feces and cecal discharges.

## MATERIALS AND METHODS

### Morphological Aspects of Broiler Feces

**Figure 1:**
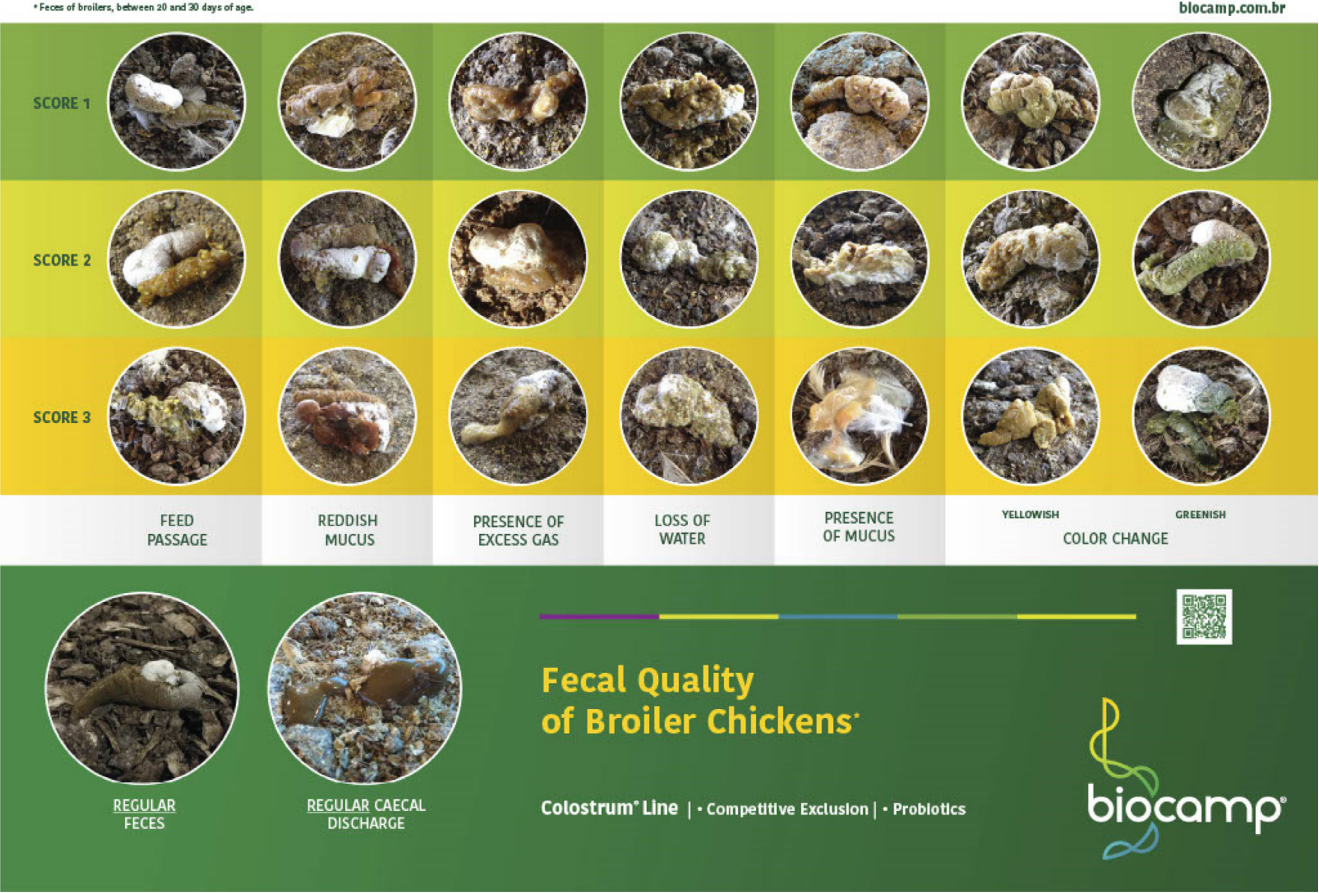
Poster of Fecal Quality of Broilers developed by Biocamp Laboratórios Ltda., which is available on the website https://biocamp.com.br/en/technical-articles/evaluation-of-faecal-quality-of-broiler-chickens/. According to authors´ right this figure may be republished anytime by the authors.

Broiler ileal feces can show several morphological alterations, in a single appearance or associated with several factors. This study was conducted according to Alvarenga (2020) and the parameters were based on morphological change, such as discrete, moderate, or intense aspects of feces (Figure 1), which illustrates the morphological differences between normal ileal feces and ileal feces with morphological changes such as feed passage, reddish mucus, watery content, the presence of mucus and foamy content, and color changes (yellowish and greenish). Each type of morphological alteration is classified into different levels according to its intensity: score 1 (slight), score 2 (moderate), and score 3 (intense).

### Study Description

The described study was performed in accordance with the Ethics Committee on Animal Use (CEUAx) of the University of São Paulo, number 8193090819. Fresh fecal samples were collected from a commercial, mixed flock of 36,000 broilers of the Ross AP95 strain, at 19 d of age, housed in a dark house in the state of São Paulo, Brazil. The litter was remounted with peanut shell. Each group was composed of 6 samples, totaling 48 samples. In Groups 1, 2, and 3, ileal feces with feed passage (**FP**) were analyzed with scores of 1, 2, and 3, respectively. In Groups 4, 5, and 6, ileal feces with reddish mucus (**RM**) were analyzed at scores of 1, 2, and 3, respectively. Group 7 analyzed normal ileal feces (**NIF**), and Group 8 analyzed cecal discharges (**CD**). The variations in the morphological appearance of feces, studied in Groups 1 to 6, were divided into 3 levels: score 1 (slight); score 2 (moderate), and score 3 (intense), according to the Poster of Fecal Quality of Broilers (Figure 1).

### Nutritional Program

The feed was formulated based on corn and soybean meal, according to the nutritional suggestions adopted by the company (Supplementary data 1 and 2).

The probiotic Colostrum Mix (Biocamp Laboratories Ltda., Campinas, Brazil) was used until the 21st day of age. This probiotic is classified as Normal Avian Gut Flora (N.A.G.F. because it is an undefined microbiota product composed of total anaerobic bacteria (1.0 × 10^4.0^ CFU/g), lactic acid-producing bacteria (1.0 × 10^4.0^ CFU/g) and mannan oligosaccharides (370 g/kg).

Aiming at the best development of the broilers, the adopted feeding program was composed of 4 pelleted diets. The prestarter diet was fed between D 1 and 7, the starter diet between D 8 and 21, the grower diet between D 22 and 34, and the final diet from D 35 until the birds were slaughtered.

### Sample Collection

**Figure 2:**
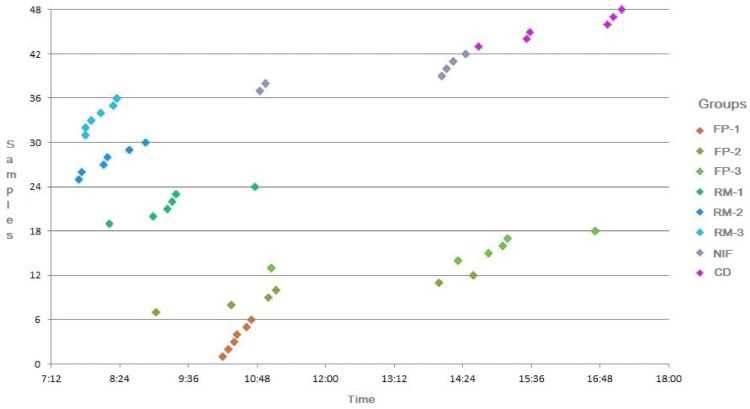
Scatter of the fecal samples collected during the 19th day of life from broilers. Group 1: FP-1; Group 2: FP-2; Group 3: FP-3; Group 4: RM-1; Group 5: RM-2; Group 6: RM-3; Group 7: NIF and Group 8: CD.

The 48 fresh fecal samples were collected on a single day, the first at 7:41 am and the last at 5:11 pm (Figure 2).

The samples were collected aseptically using a stainless-steel spatula with a spoon––both sterilized––targeting the surface and center of the feces, thus avoiding contact with litter and/or the presence of uric acid.

Sterile 1.5 mL microtubes were used to package the samples, which did not exceed 0.5 mL per sample.

The samples were immediately packed in a thermal box containing dry ice and transported to the laboratory where they were processed.

### DNA Extraction and Library Preparation

DNA extraction was performed using the MagMAX CORE kit (Life Technologies Corporation, TX) and a previous step of mechanical lysis with glass beads, according to the manufacturer's adapted protocol. The quality of the extracted DNA was evaluated by agarose gel electrophoresis. Then, the first PCR was performed for amplification of the V4 region of the 16S rRNA gene with forward (5′-TCGTCGGCAGCGTCAGATGTGTATAAGACAG-3′) and reverse (5′-GTCTCGTGGCTCGGAGATGTGTATAAGACAG-3′) primer pairs using a King Fischer thermal cycler.

Afterward, AMPure XP magnetic beads (Beckman Coulter Life Sciences, Indianapolis, IN) were used for product purification and PCR according to the manufacturer's recommendation. The size of the amplicons generated in the PCR was evaluated by agarose gel electrophoresis.

A second PCR was performed to ligate the Nextera XT kit (Illumina, CA) barcodes and confect the DNA libraries. Subsequently, the libraries were quantified to ensure the composition of a sample pool with equimolar amounts of each library. To introduce complexity to the sequencing, a heterogeneous control, the phage PhiX, was combined with the pool of amplicons. Finally, denaturation of the libraries and PhiX was performed to allow for sequencing. The libraries were sequenced using Illumina MiSeq (Illumina, CA). The depth was 100,000 sequencing reads.

### Analysis of Sequencing Data

The DADA2 program, an open package implemented in the R language (Callahan et al., 2015), was used for modeling and error correction of amplicons without the construction of operational taxonomic units (OTUs). The DADA2 package has a complete pipeline implemented to transform sequencer fastq files into inferred, dismembered, nonchimeric sample sequences. Filtering of fastq files was performed to cut primer sequences, filtering the ends due to quality drop (Q < 30). Filtering removed 5 bp from each end of the forward and reverse reads, maintaining the overlap for subsequent joining of the reads and reassembly of the V4 region fragment. A dereplication (denoizing) step was performed to obtain a detailed list of unique sequences and their abundances and produce consensus position quality scores for each unique sequence by taking the average of the positional qualities of the component reads. Since fusion occurs after the denoizing step, exact overlap is needed, with no mismatches, since substitution errors and chimeras have already been removed.

After initial data processing, taxonomies were assigned to each ASV (amplicon sequencing variant) using a DADA2 program implementation of the naive Bayesian classification method developed for this purpose. The assign Taxonomy function takes as input a set of sequences (ASVs) to be classified and a training set of reference sequences with known taxonomy to assign the taxonomies. The Silva 132 database was used as a reference.

The data generated by the DADA2 program were imported into the Phyloseq program (Murdie and Holmes, 2013). The Phyloseq package is a tool to import, store, analyze and graphically display complex phylogenetic sequencing data that can be grouped into ASVs. This package takes advantage of many of the tools available in R for ecology and phylogenetic analysis (vegan, ade4, ape, picante) while using advanced/flexible graphing systems (ggplot2) to produce graphs. Alpha and beta diversity analyses were performed in the Phyloseq package (Callahan et al., 2015). Next, ASVs that were not classified to the family level were filtered out. ASVs flagged as the same species were clustered together.

### Statistical Analysis

The Phyloseq file with the taxonomy counts was imported into the edgeR program (Robinson et al., 2010), a package of R/Bioconductor (Gentleman et al., 2004). For differential abundance analysis between groups and the analysis of continuous data, the limma voom packages were used for normalization, along with edgeR. Data visualization was performed in a BioinfoNGS program developed by the company NGS Genomic Solutions.

Analysis of variance (ANOVA) was the statistical model used in this descriptive study. To compare alpha diversity between groups, the F Test was used. Finally, Student's t test was used to analyze the relative abundance of phyla and genera, where *P* values less than 0.01 were considered statistically different.

## RESULTS AND DISCUSSION

**Figure 3:**
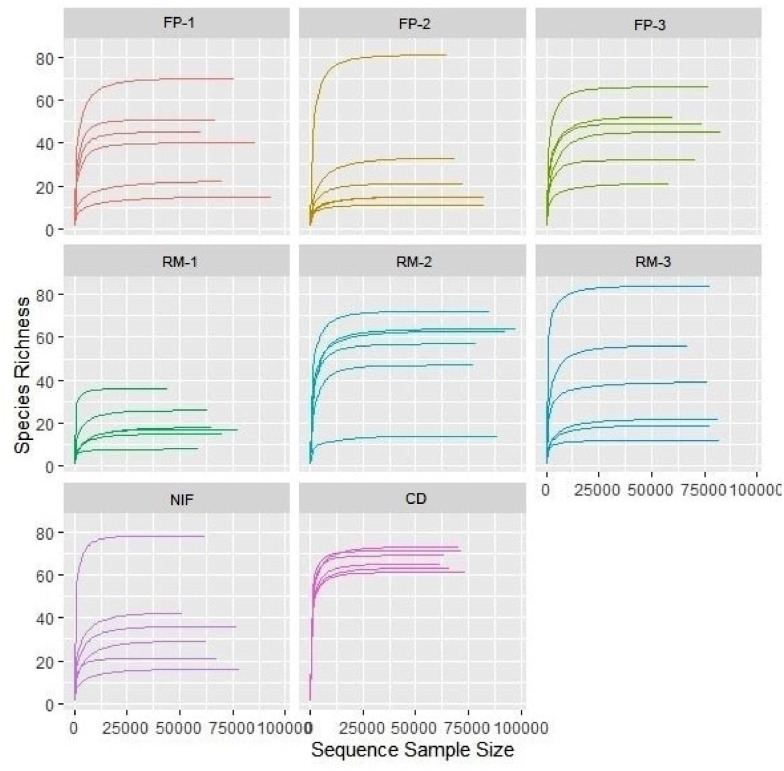
Rarefaction curves of bacterial communities representing different types and aspects of broiler feces at 19 days of age. Rarefaction curves calculated according to region V4 of the 16S rRNA gene of the different groups studied. Group 1: FP-1; Group 2: FP-2; Group 3: FP-3; Group 4: RM-1; Group 5: RM -2; Group 6: RM -3; Group 7: NIF and Group 8: CD.

The rarefaction curves attest that all DNA sequencing steps occurred successfully. This is evidenced by the formation of a long stability plateau (Figure 3).

The overall average coverage of the samples was 72,997 reads, with the lowest and highest coverage of 44,039 and 97,502 reads, respectively.

**Figure 4:**
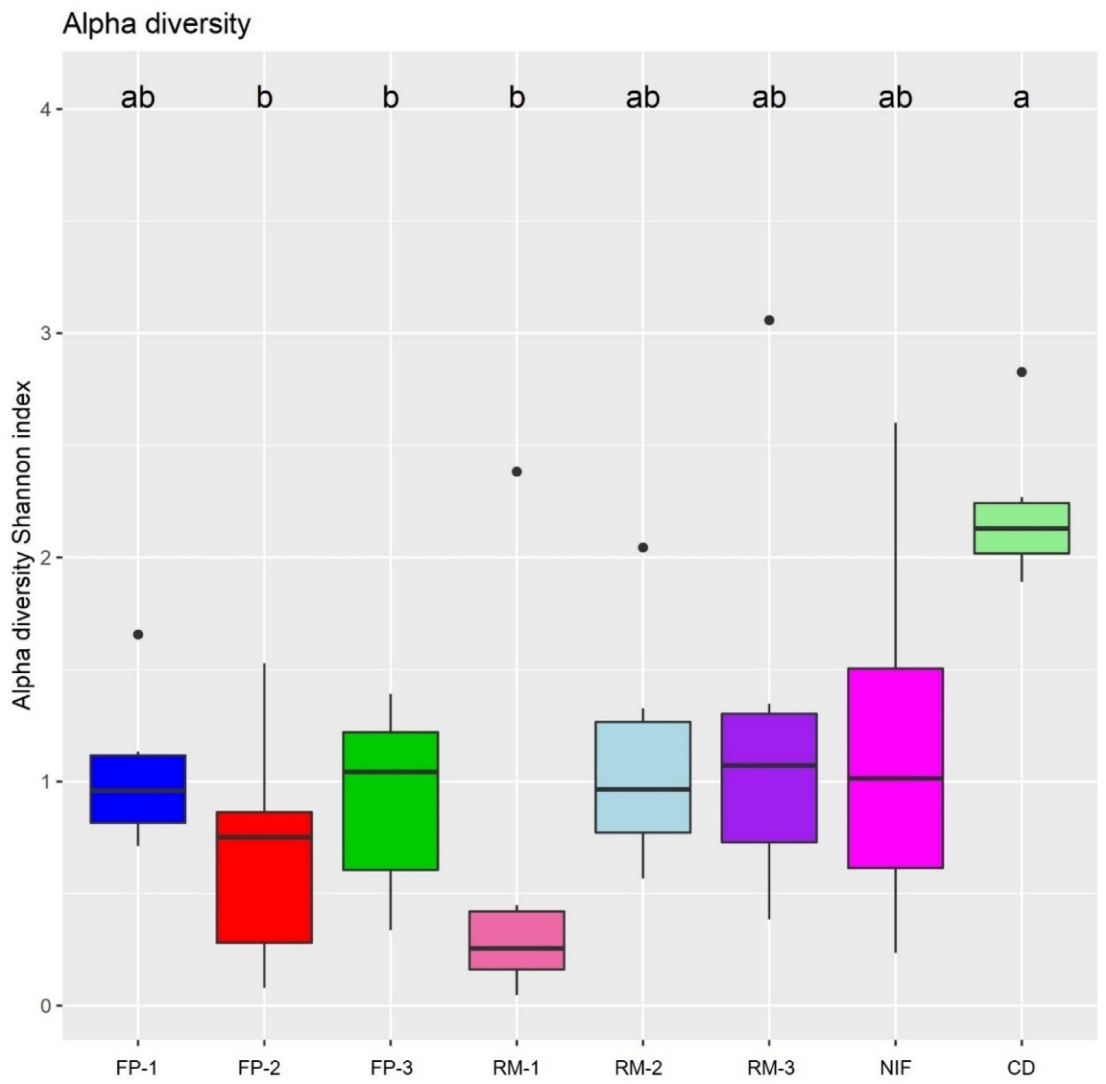
Boxplots represent the Shannon index alpha diversity of the studied groups. Group 1: FP-1; Group 2: FP -2; Group 3: FP -3; Group 4: RM-1; Group 5: RM-2; Group 6: RM-3; Group 7: NIF and Group 8: CD. Different letters indicate statistical difference (ANOVA and F Test, *P* < 0.05).

The bacterial richness present in each group was evaluated by the alpha Shannon index diversity and revealed significant differences among the groups analyzed (Figure 4).

The results indicate that the CD group had the highest bacterial richness and was significantly different from the FP-2, FP-3 and RM-1 groups. FP-1, RM-2, RM-3, and NIF were like each other and to the other groups.

**Figure 5:**
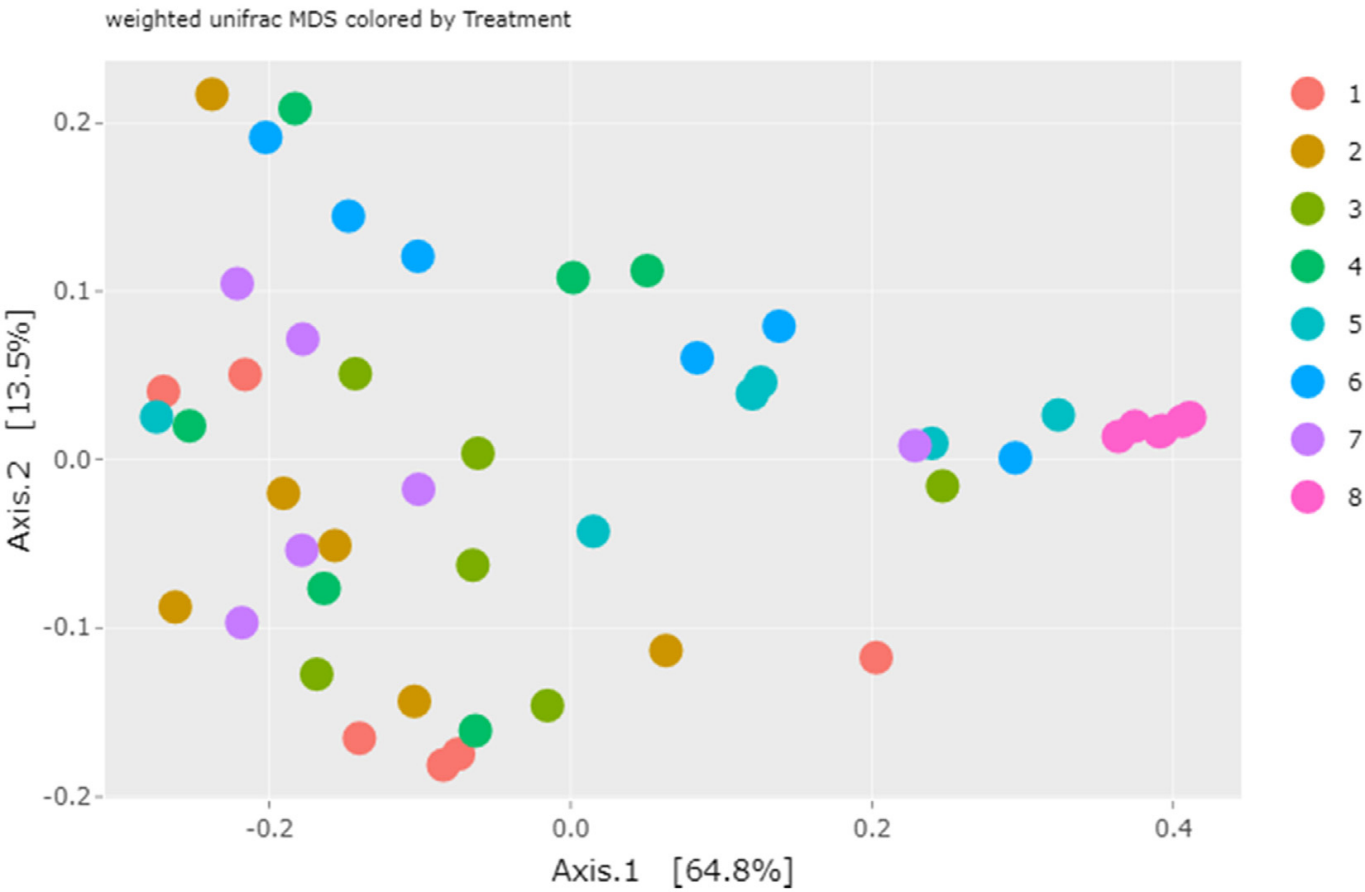
UniFrac weighted distance (MDS) illustrates the phylogenetic distances among the samples and their groups. Group 1: FP-1; Group 2: FP-2; Group 3: FP-3; Group 4: RM-1; Group 5: RM-2; Group 6: RM-3; Group 7: NIF and Group 8: CD.

The beta diversity is a measure of the heterogeneity of the bacterial communities in the samples and is represented by the multidimensional scaling (MDS) plot, as in Figure 5, which shows the weighted UniFrac distance metric.

Notably, there was great similarity among the groups, with the CD group presenting an even greater homogeneity of its samples. This reveals that the differences between the groups formed by ileal stool samples and cecal discharges are more evident.

### Relative Abundance of the Phylum and Genera in NIF and FP (1, 2 and 3)

**Figure 6:**
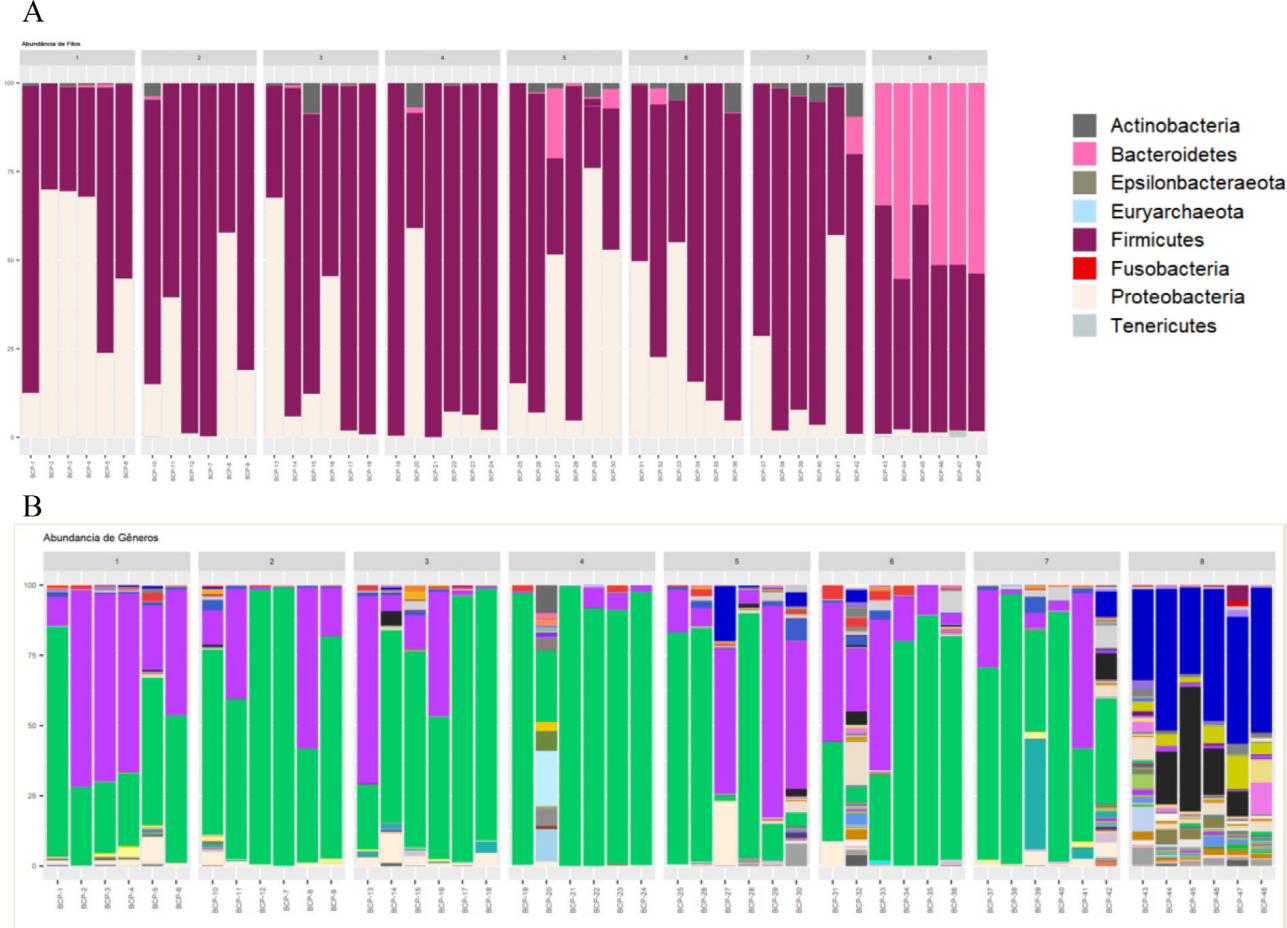
Composition of bacterial communities at the phylum level, present in ileal feces and cecal discharges of broilers, at 19 days of age. Group 1: FP-1; Group 2: FP-2; Group 3: FP-3; Group 4: RM-1; Group 5: RM-2; Group 6: RM-3; Group 7: NIF and Group 8: CD.

When comparing the relative abundance of the phyla found in the NIF group with those found in the FP-1, FP-2, and FP-3 groups, *Firmicutes* and *Proteobacteria* were the most abundant phyla (Figure 6 and Table 1).

**Table 1:** Phylum-level relative abundance of bacterial communities present in ileal feces and cecal discharges of broilers at 19 d of age.

Phylum	FP-1	FP-2	FP-3	RM-1	RM-2	RM-3	NIF	CD
*Actinobacteria*	0.52%	0.75%	1.87%	1.32%	1.90%	2.57%	3.50%	0.05%
*Bacteroidetes*	0.29%	0.15%	0.23%	0.30%	4.60%	0.84%	1.80%	**46.74%**
*Euryarchaeota*	0.04%	0.01%	0.01%	0.00%	0.00%	0.00%	0.02%	0.00%
*Epsilonbacteraeota*	0.00%	0.01%	0.01%	0.00%	0.01%	0.00%	0.00%	0.00%
*Firmicutes*	**51.06%**	**76.93%**	**75.54%**	**85.81%**	**54.15%**	**70.20%**	**78.02%**	**51.62%**
*Fusobacteria*	0.01%	0.01%	0.00%	0.00%	0.00%	0.00%	0.00%	0.00%
*Proteobacteria*	**48.07%**	**22.09%**	**22.33%**	**12.56%**	**39.35%**	**26.38%**	**16.65%**	1.16%
*Tenericutes*	0.02%	0.04%	0.02%	0.00%	0.00%	0.00%	0.01%	0.44%
***Total***	**100.00%**	**100.00%**	**100.00%**	**100.00%**	**100.00%**	**100.00%**	**100.00%**	**100.00%**

Group 1: FP-1; Group 2: FP-2; Group 3: FP-3; Group 4: RM-1; Group 5: RM-2; Group 6: RM-3; Group 7: NIF and Group 8: CD.

**Figure 7:**
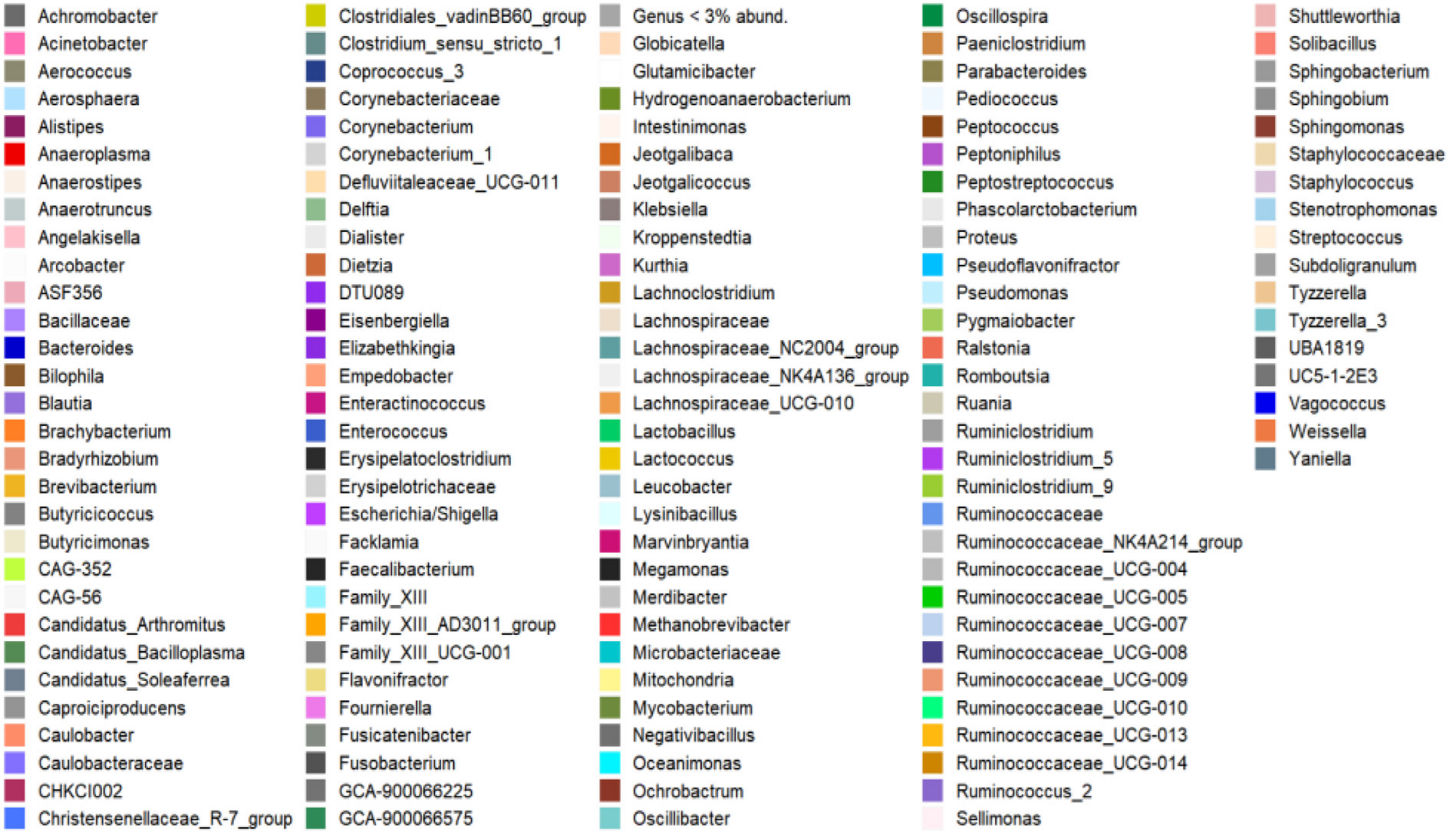
Composition of bacterial communities at the genus level, present in ileal feces and cecal discharges of broilers, at 19 days of age. Group 1: FP-1; Group 2: FP-2; Group 3: FP-3; Group 4: RM-1; Group 5: RM-2; Group 6: RM-3; Group 7: NIF and Group 8: CD.

The most abundant genera in all groups were *Lactobacillus* and *Escherichia/Shigella*. The exception was group FP-1, where an inversion of the abundance of these 2 genera was observed (Figure 7 and Table 2).

**Table 2:** Genus-level relative abundance of bacterial communities present in ileal feces and cecal discharges of broilers at 19 d of age.

Genus	FP-1	FP-2	FP-3	RM-1	RM-2	RM-3	NIF	CD
*Bacteroides*	0.30%	0.10%	0.20%	0.04%	4.20%	0.80%	1.60%	**42.90%**
*Escherichia/Shigella*	**46.30%**	**21.10%**	**21.80%**	2.70%	**38.10%**	**26.00%**	**15.60%**	1.00%
*Faecalibacterium*	0.10%	0.10%	1.00%	0.02%	0.70%	0.90%	1.60%	**14.80%**
*Lactobacillus*	**44.30%**	**73.10%**	**66.00%**	**83.50%**	**37.90%**	**53.30%**	**59.90%**	0.60%
*Outros*	**9.00%**	**5.60%**	**11.00%**	**13.74%**	**19.10%**	**19.10%**	**21.30%**	**40.70%**
***Total***	**100,00%**	**100,00%**	**100,00%**	**100,00%**	**100,00%**	**100,00%**	**100,00%**	**100,00%**

Group 1: FP-1; Group 2: FP-2; Group 3: FP-3; Group 4: RM-1; Group 5: RM-2; Group 6: RM-3; Group 7: NIF and Group 8: CD.

Six genera showed significantly affected abundance (*P* < 0.01) when comparing the NIF group with the FP-1, FP-2, and FP-3 groups (Table 3, Table 4, Table 5). The genera *Corynebacterium, Corynebacterium_1, Facklamia, Globicatella*, and *Staphylococcus* were more abundant in NIF. Only *Candidatus savagella*, formerly called *Candidatus arthromitus*, was more abundant in the FP-1 group. This shows that there is variation between the bacterial communities that make up the microbiota of normal and feed-passed ileal feces. According to Liao et al. (2012), the genus *Candidatus savagella* is present only in the ileal mucosa and cannot be detected in the cecum. This finding corroborates our study, since *Candidatus savagella* was not detected in the samples in the DC group. On the other hand, the NIF group showed a greater abundance of the genus *Corynebacterium*.

**Table 3:** Microorganisms that were significantly different (*P* < 0.01) between the NIF and FP-1 groups of fresh feces samples from broilers at 19 d of age.

Kingdom	Phylum	Class	Order	Family	Genus	logFC	P Value
*Bacteria*	*Actinobacteria*	*Actinobacteria*	*Corynebacteriales*	*Corynebacteriaceae*	*Corynebacterium*	238.561.016.783.835	0.0029
*Bacteria*	*Firmicutes*	*Bacilli*	*Lactobacillales*	*Aerococcaceae*	*Facklamia*	309.557.953.228.142	0.0063
*Bacteria*	*Firmicutes*	*Bacilli*	*Lactobacillales*	*Aerococcaceae*	*Globicatella*	241.382.179.660.352	0.0068
*Bacteria*	*Firmicutes*	*Clostridia*	*Clostridiales*	*Clostridiaceae_1*	*Candidatus Savagella*	-431.581.536.897.696	0.0074

A positive logFC indicates a higher abundance in NIF, and a negative logFC indicates a higher abundance in FP-1.

**Table 4:** Microorganisms that were significantly different (*P* < 0.01) between the NIF and FP-2 groups of fresh feces samples from broilers at 19 d of age.

Kingdom	Phylum	Class	Order	Family	Genus	logFC	P Value
*Bacteria*	*Actinobacteria*	*Actinobacteria*	*Corynebacteriales*	*Corynebacteriaceae*	*Corynebacterium_1*	436.855.636.944.741	0.0011
*Bacteria*	*Firmicutes*	*Bacilli*	*Bacillales*	*Staphylococcaceae*	*Staphylococcus*	435.406.100.201.013	0.0021
*Bacteria*	*Firmicutes*	*Bacilli*	*Lactobacillales*	*Aerococcaceae*	*Globicatella*	242.995.625.344.459	0.0064
*Bacteria*	*Actinobacteria*	*Actinobacteria*	*Corynebacteriales*	*Corynebacteriaceae*	*Corynebacterium*	217.277.274.245.482	0.0065

A positive LogFC means higher abundance in NIF.

**Table 5:** Microorganisms that were significantly different (*P* < 0.01) between the NIF and FP-3 groups of fresh feces samples from broilers at 19 d of age.

Kingdom	Phylum	Class	Order	Family	Genus	logFC	P Value
*Bacteria*	*Actinobacteria*	*Actinobacteria*	*Corynebacteriales*	*Corynebacteriaceae*	*Corynebacterium*	230.202.433.931.811	0.0039

A positive LogFC means higher abundance in NIF.

When comparing the FP-1, FP-2, and FP-3 groups, only the genus *Pediococcus* was more abundant in FP-1 than in FP-2. According to previous studies (Torok et al., 2009; Satheesh et al., 2012; Kim et al., 2016), this genus has probiotic characteristics. However, it can be said that no progressive relationship was found between the intensity levels of this type of fecal alteration and their respective microbiotas (Table 6).

**Table 6:** Microorganisms that were significantly different (*P* < 0.01) between the NIF and FP-3 groups of fresh feces samples from broilers at 19 d of age.

Kingdom	Phylum	Class	Order	Family	Genus	logFC	P Value
*Bacteria*	*Firmicutes*	*Bacilli*	*Lactobacillales*	*Lactobacillaceae*	*Pediococcus*	164.731.073.110.841	0.0092

A positive LogFC means higher abundance in NIF.

### Relative Abundance of the Phylum and Genera in NIF and RM (1, 2, and 3)

When comparing the relative abundance of the phyla found in the NIF group with those found in the RM-1, RM-2, and RM-3 groups, *Firmicutes* and *Proteobacteria* were the most abundant phyla (Figure 6 and Table 1).

Regarding the relative abundance of the genera, it was possible to identify that the genus *Lactobacillus* was the most abundant, followed by *Escherichia/Shigella*. However, this relationship was inverted in the RM-2 group (Figure 7 and Table 2).

Eleven genera had their abundances significantly changed (*P* < 0.01) when comparing NIF with the RM-1, RM-2, and RM-3 groups (Table 7, Table 8, Table 9). The genera *Brevibacterium, Brachybacterium, Clostridium_sensu_stricto_1, Corynebacterium, Corynebacterium_1, Peptostreptococcus*, and *Romboutsia* were more abundant in NIF. The genus *Stenotrophomonas* was most abundant in RM-1. The genera *Anaerostipes* and *Caproiciproducens* were most abundant in the RM-2 group. The genera *Candidatus savagella* and *Caproiciproducens* were most abundant in group RM-3. The increased relative abundance of bacterial communities forming short-chain fatty acid (**SCFA**)-producing genera (acetate, butyrate, and capric) such as *Anaerostipes* in group RM-2 and *Caproiciproducens* in groups RM-2 and RM-3 is suggestive of a more intense repair process of the intestinal epithelium, since both groups showed lower relative abundance of the genus *Lactobacillus* (37.9 and 53.3%, respectively) when compared to the NIF group, which showed an abundance of 59.9% (Table 2). On the other hand, it was possible to observe that in the RM-2 and RM-3 groups, there was an increase in the relative abundance of the *Escherichia/Shigella* genus, which may be related to the higher intensity of reddish mucus, since this bacterial genus has the metabolic ability to colonize and use intestinal mucins as a source of carbon, protein, and energy (Conway and Cohen, 2015).

**Table 7:** Microorganisms that were significantly different (*P* < 0.01) between the NIF and RM-1 groups of fresh feces samples from broilers at 19 d of age.

Kingdom	Phylum	Class	Order	Family	Genus	logFC	P Value
*Bacteria*	*Firmicutes*	*Clostridia*	*Clostridiales*	*Peptostreptococcaceae*	*Romboutsia*	66.190.103.403.794	1,95E+09
*Bacteria*	*Firmicutes*	*Clostridia*	*Clostridiales*	*Clostridiaceae_1*	*Clostridium_sensu_stricto_1*	336.577.243.235.381	0.0010
*Bacteria*	*Actinobacteria*	*Actinobacteria*	*Micrococcales*	*Brevibacteriaceae*	*Brevibacterium*	423.915.201.693.891	0.0012
*Bacteria*	*Actinobacteria*	*Actinobacteria*	*Micrococcales*	*Dermabacteraceae*	*Brachybacterium*	356.719.350.659.315	0.0040
*Bacteria*	*Actinobacteria*	*Actinobacteria*	*Corynebacteriales*	*Corynebacteriaceae*	*Corynebacterium_1*	379.142.840.625.466	0.0041
*Bacteria*	*Firmicutes*	*Clostridia*	*Clostridiales*	*Peptostreptococcaceae*	*Peptostreptococcus*	275.947.620.605.471	0.0056
*Bacteria*	*Actinobacteria*	*Actinobacteria*	*Corynebacteriales*	*Corynebacteriaceae*	*Corynebacterium*	212.871.475.447.178	0.0074
*Bacteria*	*Proteobacteria*	*Gammaproteobacteria*	*Xanthomonadales*	*Xanthomonadaceae*	*Stenotrophominas*	300.449.099.708.308	0.0098

A positive LogFC means higher abundance in NIF, and a negative logFC means higher abundance in RM-1.

**Table 8:** Microorganisms that were significantly different (*P* < 0.01) between the NIF and RM-2 groups of fresh feces samples from broilers at 19 d of age.

Kingdom	Phylum	Class	Order	Family	Genus	logFC	P Value
*Bacteria*	*Firmicutes*	*Clostridia*	*Clostridiales*	*Lachnospiraceae*	*Anaerostipes*	-400.649.748.138.595	0.0004
*Bacteria*	*Actinobacteria*	*Actinobacteria*	*Corunebacteriales*	*Corynebacteriaceae*	*Corynebacterium*	260.714.535.621.945	0.0012
*Bacteria*	*Firmicutes*	*Clostridia*	*Clostridiales*	*Clostridiaceae_1*	*Clostridium_sensu_stricto_1*	327.733.427.477.228	0.0021
*Bacteria*	*Firmicutes*	*Clostridia*	*Clostridiales*	*Ruminococcaceae*	*Caproiciproducens*	-251.586.564.639.376	0.0037
*Bacteria*	*Firmicutes*	*Clostridia*	*Clostridiales*	*Peptostreptococcaceae*	*Peptostreptococcus*	282.730.154.916.408	0.0058

A positive LogFC means higher abundance in NIF, and a negative logFC means higher abundance in RM-2.

**Table 9:** Microorganisms that were significantly different (*P* < 0.01) between the NIF and RM-3 groups of fresh feces samples from broilers at 19 d of age.

Kingdom	Phylum	Class	Order	Family	Genus	logFC	*P* value
*Bacteria*	*Firmicutes*	*Clostridia*	*Clostridiales*	*Peptostreptococcaceae*	*Romboutsia*	672.026.415.934.395	1,54E + 09
*Bacteria*	*Firmicutes*	*Clostridia*	*Clostridiales*	*Clostridiaceae_1*	*Candidatus Savagella*	-521.859.073.864.715	0.0006
*Bacteria*	*Firmicutes*	*Clostridia*	*Clostridiales*	*Peptostreptococcaceae*	*Peptostreptococcus*	307.323.447.608.459	0.0022
*Bacteria*	*Firmicutes*	*Clostridia*	*Clostridiales*	*Ruminococcaceae*	*Caproiciproducens*	-257.282.650.909.226	0.0029
*Bacteria*	*Firmicutes*	*Clostridia*	*Clostridiales*	*Clostidiaceae_1*	*Clostridium_sensu_stricto_1*	294.020.782.488.166	0.0059

A positive LogFC means higher abundance in NIF, and a negative logFC means higher abundance in RM-3.

When comparing the RM-1, RM-2, and RM-3 groups with each other, fourteen genera showed significant differences in their relative abundances. Comparison of the RM-1 and RM-2 groups revealed that *Anaerostipes, Brachybacterium* and *Butyricicoccus* were more abundant in RM-2 (Table 10). When comparing RM-1 with RM-3, *Achromobacter, Caulobacter, Elizabethkingia, Delftia, Dialister, Sphingobium, Sphingomonas*, and *Stenotrophomonas* were more abundant in RM-1, while *Brachybacterium, Caproiciproducens* and *Escherichia/Shigella* were more abundant in RM-3 (Table 11). Finally, when comparing groups RM-2 and RM-3, only the genus *Corynebacterium* was more abundant in RM-3 than in RM-2 (Table 12).

**Table 10:** Microorganisms that were significantly different (*P* < 0.01) between the RM-1 and RM-2 groups of fresh feces samples from broilers at 19 d of age.

Kingdom	Phylum	Class	Order	Family	Genus	logFC	P Value
*Bacteria*	*Firmicutes*	*Clostridia*	*Clostridiales*	*Lachnospiraceae*	*Anaerostipes*	-392.365.602.170.899	0.0005
*Bacteria*	*Firmicutes*	*Clostridia*	*Clostridiales*	*Ruminococcaceae*	*Butyricicoccus*	-503.492.173.601.261	0.0007
*Bacteria*	*Actinobacteria*	*Actinobacteria*	*Micrococcales*	*Dermabacteraceae*	*Brachybacterium*	-418.188.733.397.555	0.0008

A negative logFC means a higher abundance in RM-2.

**Table 11:** Microorganisms that were significantly different (*P* < 0.01) between the NIF and RM-3 groups of fresh feces samples from broilers at 19 d of age.

Kingdom	Phylum	Class	Order	Family	Genus	logFC	P Value
*Bacteria*	*Proteobacteria*	*Gammaproteobacteria*	*Enterobacteriales*	*Enterobacteriales*	*Escherichia/Shigella*	-393.602.343.838.278	0.0023
*Bacteria*	*Proteobacteria*	*Alphaproteobacteria*	*Sphingomonadales*	*Sphingomonadaceae*	*Sphingomonas*	341.283.729.107.065	0.0033
*Bacteria*	*Proteobacteria*	*Alphaproteobacteria*	*Caulobacterales*	*Caulobacteraceae*	*Caulobacter*	350.455.793.536.553	0.0036
*Bacteria*	*Firmicutes*	*Clostridia*	*Clostridiales*	*Ruminococcaceae*	*Caproiciproducens*	-24.899.850.494.153	0.0039
*Bacteria*	*Proteobacteria*	*Gammaproteobacteria*	*Xanthomonadales*	*Xanthomonadaceae*	*Stenotrophomonas*	3.235.407.807.436	0.0056
*Bacteria*	*Bacteroidetes*	*Bacteroidia*	*Flavobacteriales*	*Weeksellaceae*	*Elizabethkingia*	219.552.244.732.337	0.0063
*Bacteria*	*Proteobacteria*	*Gammaproteobacteria*	*Betaproteobacteriales*	*Burkholderiaceae*	*Achromobacter*	28.276.311.461.357	0.0064
*Bacteria*	*Proteobacteria*	*Alphaproteobacteria*	*Sphingomonadales*	*Sphingomonadaceae*	*Sphingobium*	269.818.328.446.598	0.0067
*Bacteria*	*Proteobacteria*	*Gammaproteobacteria*	*Betaproteobacteriales*	*Burkholderiaceae*	*Delftia*	231.030.458.211.489	0.0072
*Bacteria*	*Firmicutes*	*Negativicutes*	*Selenomonadales*	*Veillonellaceae*	*Dialister*	193.341.195.670.231	0.0075
*Bacteria*	*Actinobacteria*	*Actinobacteria*	*Micrococcales*	*Microbacteriaceae*	*NA*	173.618.548.211.534	0.0089
*Bacteria*	*Actinobacteria*	*Actinobacteria*	*Micrococcales*	*Dermabacteraceae*	*Brachybacterium*	-31.965.839.560.586	0.0095

A positive LogFC means higher abundance in NIF, and a negative logFC means higher abundance in RM-3.

**Table 12:** Microorganisms that were significantly different (*P* < 0.01) between groups RM-2 and RM-3 extracted from fresh broiler feces samples at 19 d of age.

Kingdom	Phylum	Class	Order	Family	Genus	logFC	P Value
*Bacteria*	*Actinobacteria*	*Actinobacteria*	*Corynebacteriales*	*Corynebacteriaceae*	*Corynebacterium*	-185.015.407.120.271	0.0098

A negative logFC indicates a higher abundance in RM-3.

Beneficial genera such as *Anaerostipes, Butyricicococcus*, and *Caproiciproducens* are related to the synthesis of butyrate (Le Gall et al., 2009; Ahmed et al., 2014; Ritzi et al., 2014; Kim et al., 2015; Bengelsdorf et al., 2019; Shetty et al., 2020) and are responsible for stimulating the proliferation and differentiation of intestinal mucosal cells. In addition, the genus *Caproiciproducens* also synthesizes capric acid, which has a spectrum of action against gram-negative pathogenic bacteria (Hinton Jr and Ingram, 2011). This variation in abundance may be an attempt to reestablish eubiosis and intestinal integrity.

### Relative Abundance of the Phylum and Genera in NIF and DC

When comparing the NIF and DC groups, the most abundant phyla were *Firmicutes* (78.0%) and *Proteobacteria* (16.6%) in the NIF group. In the CD group, the abundance of *Firmicutes* and *Bacteroidetes* were 51.6% and 46.7%, respectively (Table 1). The data in our study corroborate several previous studies (Nordentoft et al., 2011; Oakley et al., 2014; Bae et al., 2017; Siegerstetter et al., 2018; Glendinnig et al., 2019; Hong, et al., 2019; Kollarcikova et al., 2019; Shah et al., 2019; Shi et al., 2019). On the other hand, 2 studies presented different data. Yan et al. (2017) analyzed the intestinal contents (duodenum and caeca) and feces of broiler chickens and found that in both cases, *Firmicutes* and *Bacteroidetes* were the most abundant phyla. In the second study, Chen et al. (2019) studied the cecal contents of 28-day-old chickens, and the most abundant phyla were *Bacteroidetes* and *Firmicutes*. This shows a reversal of abundance among the main phyla that make up the cecal microbiota of broilers.

Regarding the abundance of genera, *Lactobacillus* (59.9%) and *Escherichia/Shigella* (15.6%) were the most abundant in NIF, while *Bacteroides* (42.9%) and *Faecalibacterium* (14.8%) were the most abundant in CD (Table 2). Moreover, 61 of them showed significant differences (*P* < 0.01). Such differences can be visualized in the alpha (Figure 4) and beta (Figure 5) diversity analyses. The phylogenetic distances between the groups were clearly present, since the samples of the DC group were more homogeneous, corroborating Glendinning et al. (2019), in addition to presenting a higher diversity of genera than the NIF group, corroborating previously published studies (Videnska et al., 2013; Shaufi et al., 2015; Borda-Molina et al., 2016; Munyaka et al., 2016; Ranjitkar et al., 2016; Siegerstetter et al., 2017; Wang et al., 2017; Rychlik, 2020).

Although *Firmicutes* was the most abundant phylum in the NIF and DC groups, it is possible to observe a difference between them in the most abundant bacterial genera, as noted by Kollarcikova et al. (2019).

### Regarding the Dispersion of Samples and Respective Collection Times

At 19 d of age, the adopted light program provided 18 h of light with an illuminance of 10 lux and 6 h of dark (Annex 3). The dark period started at 00:00 am and ended at 06:00 am. Thus, the first sample was collected 1 h 41 min after the lights were turned on. It is known that during the dark period, broilers reduce their activity and consume less feed (Schwean-Lardner and Classen, 2010), leaving their digestive tract with less feed (Ortigues and Doreau, 1995).

After collecting the samples, it was possible to observe a relationship between some groups and the time of collection, which may be a result of the light program adopted (Figure 2).

Groups RM-1, RM-2, and RM-3 were the first to have their samples collected, and all were obtained in the morning, between 7:41 am and 10:46 am, which shows that longer periods of darkness result in less feed in the intestinal tract, increasing mucus production and, therefore, modification of the microbiota (Thaiss et al., 2016; Wang et al., 2018; Metzler-Zebeli et al., 2019). The samples were collected from the FP-1, FP-2, and FP-3 groups throughout the day between 9:02 am and 4:43 pm. The six samples from the NIF group were collected between late morning and early afternoon (10:51 am to 2:27 pm), suggesting that after the regularization of feed consumption with probiotic, the intestinal microbiota was modulated, the intestinal transit was normalized and the broilers began to eliminate better quality feces, mostly without any type of alteration.

Finally, the CD group had their samples collected only in the afternoon, between 14:41 and 17:11 (Figure 2), data that corroborate with Ito et al. (2009), who published that cecal discharges are usually eliminated twice a day and always in the presence of light.

Under the conditions exposed in this study, it is possible to conclude that evaluation of fecal morphology is a fundamental task, as the morphological aspects of the feces may be related to the composition and structure of fecal microbiota. A better understanding of how the intestinal microbiota composition can affect fecal morphology will provide meaningful tools to act quickly and assertively to modulate the microbiota through the use of probiotics, favoring intestinal integrity and zootechnical gain.

## APPENDIX

The appendix will be available on the journal's website.

## Appendix Supplementary materials

Image, application 1
