# Supplementary material for: Metagenomics analysis of the morphological aspects and bacterial composition of broiler feces
Source: Poult Sci. 2022 Dec 9;102(2):102401. doi: 10.1016/j.psj.2022.102401 (PMC9800314; doi:10.1016/j.psj.2022.102401)
Supplement: Supplementary file 1 [file mmc1.docx]

| ***INGREDIENTS*** | | | | | | Cost ( R$/kg ) | | Pre-Starter | | Starter | | Grower | Finisher | | |
| --- | --- | --- | --- | --- | --- | --- | --- | --- | --- | --- | --- | --- | --- | --- | --- |
| ***Macro*** | | | ***Ingredients*** | | |  | |  | |  | |  |  | | |
| 8006 | | | Corn | | | R$534,0000 | | 1.083,2300 | | 1.191,6900 | | 1.391,7990 | 1.448,3170 | | |
| 8301 | | | Soybean meal | | | 1.180,0000 | | 737,0000 | | 641,0000 | | 360,0000 | 314,0000 | | |
| 8457 | | | Meat meal | | | 900,0000 | | 85,2000 | | 73,3000 | | 50,9000 | 41,4000 | | |
| 8552 | | | Feather meal | | | 1.200,0000 | |  | |  | | 40,0000 | 40,0000 | | |
| 8556 | | | Vicera meal | | | 1.450,0000 | |  | |  | | 60,0000 | 60,0000 | | |
| 8681 | | | Vegetable oil | | | 2.470,0000 | | 43,5700 | | 45,6600 | | 49,9510 | 51,5830 | | |
| 8815 | | | Limestone 38% | | | 210,0000 | | 9,2000 | | 8,2000 | | 7,5000 | 5,6000 | | |
| ***Micro Ingredients*** | | | | | | | | | | | | | | | |
| 8701 | | | Salt | | | 300,0000 | | 6,0000 | | 7,6000 | | 5,1000 | 4,6000 | | |
| 8764 | | | AdSodium | | | 1.681,0000 | | 3,8000 | | 2,0000 | | 1,8000 | 2,6000 | | |
| 8922 | | | Ajilys 64 | | | 3.848,0000 | | 7,2500 | | 7,8000 | | 11,8500 | 11,5500 | | |
| 8925 | | | L-Threonine | | | 6.200,0000 | | 2,6000 | | 2,4000 | | 1,9000 | 1,7000 | | |
| 8952 | | | Colostrum Mix | | | 58.870,0000 | | 1,0000 | | 0,2000 | |  |  | | |
| 8961 | | | Betaine HCL 95% | | | 8.182,0000 | | 1,0000 | | 1,0000 | | 1,0000 | 1,0000 | | |
| 8998 | | | Adsorbent | | | 4.000,0000 | | 3,0000 | | 3,0000 | | 3,0000 | 3,0000 | | |
| 9342 | | | MaxiMOS | | | 4.150,0000 | | 1,0000 | | 1,0000 | | 1,0000 | 1,0000 | | |
| ALIMET | | | Alimet | | | 7.920,0000 | | 8,1500 | | 7,1500 | | 6,2000 | 5,6500 | | |
| T15963 | | | PX VM STARTER 4KG/T R | | | 21.027,0000 | | 8,0000 | | 8,0000 | |  |  | | |
| T15964 | | | PX VM GROWER 4KG/T R | | | 14.560,0000 | |  | |  | | 8,0000 |  | | |
| T15965 | | | PX VM FINISHER 4KG/T R | | | 10.530,0000 | |  | |  | |  | 8,0000 | | |
| **Total** | | | | ( kg ) | | **2.000,0000** | | | | **2.000,0000** | | **2.000,0000** | **2.000,0000** | | |
| **Cost** | | | | ( R$/ton ) | | 1.001.253,26 | | | | 942.350,53 | | 862.817,62 | 829.411,14 | | |
| ***NUTRITIONAL LEVELS*** | | | | Unit | | Pre-Starter | | Starter | | Grower | | | Finisherl |  |  |
| 001 | UMIDITY | | | % | | 11,4042 | | 11,4590 | | 11,3435 | | | 11,37 |  |  |
| 006 | CRUDE PROTEIN | | | % | | 24,4981 | | 22,4200 | | 19,7860 | | | 18,68 |  |  |
| 025 | ETHEREAL EXTRACTS | | | % | | 5,2447 | | 5,3986 | | 6,1941 | | | 6,28 |  |  |
| 030 | RAW FIBER | | | % | | 2,6884 | | 2,5600 | | 2,0913 | | | 2,04 |  |  |
| 040 | MINERAL MATERIAL | | | % | | 5,2243 | | 4,8348 | | 4,1098 | | | 3,75 |  |  |
| 045 | CALCIUM | | | % | | 0,9500 | | 0,8500 | | 0,7740 | | | 0,68 |  |  |
| 050 | TOTAL PHOSPHORUS | | | % | | 0,6228 | | 0,5703 | | 0,5271 | | | 0,48 |  |  |
| 051 | AVAILABLE PHOSPHORUS | | | % | | 0,5000 | | 0,4595 | | 0,4300 | | | 0,40 |  |  |
| 070 | A.M. ENERGY (CHICK) | | | KCAL/KG | | 3.050,0000 | | 3.120,0000 | | 3.279,3044 | | | 3.319,27 |  |  |
| 071 | A.M. ENERGY (BROILER) | | | KCAL/KG | | 3.050,5419 | | 3.120,5957 | | 3.280,0000 | | | 3.320,00 |  |  |
| 150 | [CHICK LYSINE DIGESTIBLE](https://scholar.google.com.br/scholar?hl=pt-BR&as_sdt=0,5&q=CHICK+LYSINE+DIGESTIBLE) | | | % | | 1,3500 | | 1,2400 | | 1,1200 | | | 1,05 |  |  |
| 351 | SODIUM | | | % | | 0,2200 | | 0,2200 | | 0,1800 | | | 0,18 |  |  |

ANNEX 1 - Formulation of the diets supplied throughout the rearing of the broilers.

|  | Age (weeks) | | | | | |
| --- | --- | --- | --- | --- | --- | --- |
|  | 1 | 2 | 3 | 4 | 5 | 6 |
| Light (h) | 23 | 18 | 18 | 18 | 18 | 18 |
| Dark (h) | 1 | 6 | 6 | 6 | 6 | 6 |
|  |  |  |  |  |  |  |
| Illuminance (lux) | 25 | 15 | 10 | 5 | 5 | 5 |
|  |  |  |  |  |  |  |
| 00:00h | Dark | Dark | Dark | Dark | Dark | Dark |
| 01:00h | Light | Dark | Dark | Dark | Dark | Dark |
| 02:00h | Light | Dark | Dark | Dark | Dark | Dark |
| 03:00h | Light | Dark | Dark | Dark | Dark | Dark |
| 04:00h | Light | Dark | Dark | Dark | Dark | Dark |
| 05:00h | Light | Dark | Dark | Dark | Dark | Dark |
| 06:00h | Light | Light | Light | Light | Light | Light |
| 07:00h | Light | Light | Light | Light | Light | Light |
| 08:00h | Light | Light | Light | Light | Light | Light |
| 09:00h | Light | Light | Light | Light | Light | Light |
| 10:00h | Light | Light | Light | Light | Light | Light |
| 11:00h | Light | Light | Light | Light | Light | Light |
| 12:00h | Light | Light | Light | Light | Light | Light |
| 13:00h | Light | Light | Light | Light | Light | Light |
| 14:00h | Light | Light | Light | Light | Light | Light |
| 15:00h | Light | Light | Light | Light | Light | Light |
| 16:00h | Light | Light | Light | Light | Light | Light |
| 17:00h | Light | Light | Light | Light | Light | Light |
| 18:00h | Light | Light | Light | Light | Light | Light |
| 19:00h | Light | Light | Light | Light | Light | Light |
| 20:00h | Light | Light | Light | Light | Light | Light |
| 21:00h | Light | Light | Light | Light | Light | Light |
| 22:00h | Light | Light | Light | Light | Light | Light |
| 23:00h | Light | Light | Light | Light | Light | Light |

ANNEX 2 - Light program adopted for the creation of the plot.
